# Perspectives of physical activity in combating metabolic syndrome: insights from a multi-ethnic urban population

**DOI:** 10.3389/fpubh.2025.1477025

**Published:** 2025-08-22

**Authors:** Bushra Ali, Tim Evans, Rhonda Cohen, Anne Elliott

**Affiliations:** ^1^Department Science and Technology, London Sports Institute, Middlesex University London, London, United Kingdom; ^2^Business and Political Economy, Middlesex University London, London, United Kingdom

**Keywords:** physical activity, metabolic syndrome, Hofstede cultural framework, stakeholder theory, non-communicable diseases

## Abstract

The United Arab Emirates is experiencing a rising burden of non-communicable diseases, particularly metabolic syndrome (MetS), driven by rapid urbanization and lifestyle changes. In Dubai's diverse population, where expatriates constitute 85% of residents, understanding perspectives on physical activity (PA) is essential for effective prevention strategies. This qualitative study explored stakeholder experiences and views on PA as a preventive measure for MetS. Semi-structured interviews were conducted with 20 stakeholders, including policy officials, gym owners, trainers, gym members, and inactive individuals, recruited through purposive and snowball sampling. Data were analyzed using thematic analysis and Hofstede's cultural framework to examine cultural influences on PA behaviors. Results indicated broad recognition of PA's importance in managing MetS but identified significant cultural and environmental barriers to participation. Key themes included cultural perceptions shaping engagement with PA and infrastructure challenges. These findings highlight the need for culturally tailored interventions and multisectoral collaboration to promote active lifestyles in Dubai's multi-ethnic context. This research offers valuable insights to guide public health initiatives aiming to reduce the burden of MetS through culturally sensitive approaches suited to this unique urban environment.

## Background

Physical inactivity is formally defined as the failure to engage in a level of PA that is sufficient to meet the established public health guidelines. These guidelines specify a range of 150–300 min of moderate to vigorous PA, or 75–150 min of vigorous PA, or an equivalent combination of moderate-to-vigorous PA ((1)). It serves as a significant risk factor for developing multiple health conditions, including obesity, cardiovascular disease, diabetes, and musculoskeletal disorders. This phenomenon adversely affects metabolic function by reducing basal metabolic rate, promoting muscle atrophy, and impairing insulin sensitivity. These metabolic disruptions trigger adaptive responses whereby the body compensates for reduced PA through systematic metabolic adjustments. The resulting adaptation is characterized by elevated insulin and leptin secretion, diminished energy expenditure, and compromised insulin-mediated glucose regulation, establishing a pathophysiological cascade that may progress to MetS (2).

MetS is diagnosed when an individual has at least three of the following five criteria (3):

° Elevated waist circumference (For the Middle East^*^: men ≥ 94 cm, women ≥ 80 cm).
° Elevated triglycerides: ≥150 mg/dL (1.7 mmol/L), or specific treatment for this lipid abnormality.
° Reduced high-density lipoprotein (HDL) cholesterol: < 40 mg/dL (1.03 mmol/L) in men, < 50 mg/dL (1.29 mmol/L) in women, or specific treatment for this lipid abnormality.
° Elevated blood pressure: systolic ≥130 and/or diastolic ≥85 mm Hg, or treatment of previously diagnosed hypertension.
° Elevated fasting glucose: ≥100 mg/dL (5.6 mmol/L), or previously diagnosed type 2 diabetes.

^*^The International Diabetes Federation (IDF) provides waist circumference recommendations for MetS, which are uniform for women globally due to the limited availability of robust data. However, these recommendations differ slightly for men of European descent (Europids) compared to those of Asian descent. The classification of levels for Asian populations is derived from the recommendations provided by the WHO. Limited data is currently accessible for other regions; nevertheless, until new data becomes available, recommendations for Europid males are being applied to men from the Middle East, Eastern Mediterranean region, and Sub-Saharan Africa (4). This regional adaptation of diagnostic criteria is particularly relevant given that a systematic review by Mahmoud and Sulaiman (5) highlights that the high prevalence of MetS in the UAE has become a significant concern, primarily because of its association with premature mortality from NCDs. The potential mechanism that contributes to the development of these diseases is the high occurrence of physical inactivity (6).

In the pre-oil era, the inhabitants of the UAE typically led active and diversified lifestyles adapted to the region's geographical and climatic conditions. These lifestyles were characterized by activities such as trade, fishing, pearling, nomadic herding, agriculture, and traditional crafts (7). Many of these tasks were often performed manually or with the help of basic tools, which would require more physical effort compared to modern, mechanized methods. Nonetheless, over the last several decades there has been a significant improvement in living conditions and a rise in the usage of primarily mechanized technology, which has spread to all segments of Dubai's population. From this point on, a noticeable increase was discerned toward sedentary lifestyles and diminished levels of PA within the populace (8). While Dubai's development has brought about significant improvements in overall health and wellbeing, evidenced by increased life expectancy and reduced infant and maternal mortality rates, it has also given rise to new health challenges. The rapid urbanization and lifestyle changes associated with economic growth have led to an increase in obesity and NCDs, resulting in a rise in years lived with disability (YLDs) (9). The government of Dubai has shown a firm dedication to public health, led by Crown Prince HH Sheik Hamdan Bin Mohammad bin Rashid Al Maktoum, who is driving an ambitious effort to establish Dubai as a worldwide frontrunner in fitness and wellbeing (10). This forward-thinking strategy demonstrates the emirate's commitment to improving the wellbeing of its citizens and establishing new standards for promoting urban health.

This study is motivated by the evolving health landscape in the UAE, particularly regarding MetS, and recognizes the opportunity for customized PA initiatives that resonate with Dubai's multicultural population. Dubai's selection as the research focus aligns with its renowned openness to innovation and ongoing dedication to promoting active lifestyles. As a forward-thinking urban center, Dubai is uniquely positioned to potentially inspire and inform public health strategies, not only within the UAE but possibly across the broader Arab region, showcasing the potential of culturally attuned health promotion in diverse urban environments.

## Aims and objectives

### Aims

To explore stakeholders' perceptions of PA for the prevention and management of MetS, with a focus on identifying barriers, cultural influences, motivators, and opportunities for enhancing PA engagement.

### Objectives

- To examine the perceptions and experiences of stakeholders (from policy developers to end-users) regarding PA for MetS prevention and management.
- To identify key barriers, cultural influences, and motivators affecting PA engagement among stakeholders.
- To explore opportunities and strategies suggested by stakeholders for improving PA engagement and MetS prevention in the future.
- To assess stakeholders' priorities and aspirations for enhancing PA participation in the context of MetS.

## Methods

### Theoretical framework

Interpretivism underpins this study, focusing on how individuals construct meaning through social interactions (11). This paradigm is particularly relevant for exploring perspectives on PA, as these are shaped by shared assumptions and collective experiences that reveal the motivations and processes behind organizational and individual actions (12). The ontological stance is constructivist, positing that reality is not fixed but is continually created through social relationships; as Walker et al. (13) note, truth is found in social spaces and interactions. Consequently, the research question is grounded in the socially constructed views of individuals, and understanding these multiple perspectives is key to generating knowledge from a constructivist viewpoint. This interpretivist-constructivist approach is particularly suited to multicultural contexts such as Dubai, where diverse social realities must be explored to understand the PA landscape.

This study employs Stakeholder Theory as its conceptual foundation, defining stakeholders as individuals or groups affected by, interested in, or able to influence PA interventions (14, 15). Stakeholder Analysis serves as the primary qualitative methodology, systematically identifying, categorizing, and prioritizing stakeholders based on their interests, influence, and potential impact (16). This approach uniquely examines data both within and across stakeholder groups, revealing group-specific and overarching themes that capture the complexity of stakeholder perspectives in PA initiatives. Stakeholder engagement throughout the research process is essential for integrating evidence into interventions, enhancing their relevance, sustainability, transparency, and community ownership (17).

Data are collected through semi-structured interviews, a method well-suited to interpretivist research as it acknowledges the collaborative and co-constructive nature of knowledge production between researchers and participants (11). This approach allows for in-depth exploration of stakeholder experiences, needs, and motivations, providing actionable insights for the design and delivery of effective, contextually relevant PA interventions.

### Sample

Stakeholders were organized into six defined “levels” based on their functional roles within the PA ecosystem. These levels were then mapped onto the Power–Interest Matrix according to their relative influence (power) and engagement (interest) in the project. The classification was as follows:

- **Level 1: Senior Policy Officials**—High Power, Low Interest
- **Level 2: Gym Owners**—High Power, High Interest
- **Level 3: Gym Managers**—Moderate Power, High Interest
- **Level 4: Personal Trainers**—Low Power, High Interest
- **Level 5: Active Individuals/Gym Members**—Low Power, Moderate Interest
- **Level 6: Inactive Individuals**—Low Power, Low Interest

Active individuals (Level 5) were defined as those self-reporting at least 150 min of moderate physical activity per week, aligning with WHO guidelines. Inactive individuals (Level 6) reported less or no regular activity. Classification occurred during initial screening interviews.

**Figure 1:**
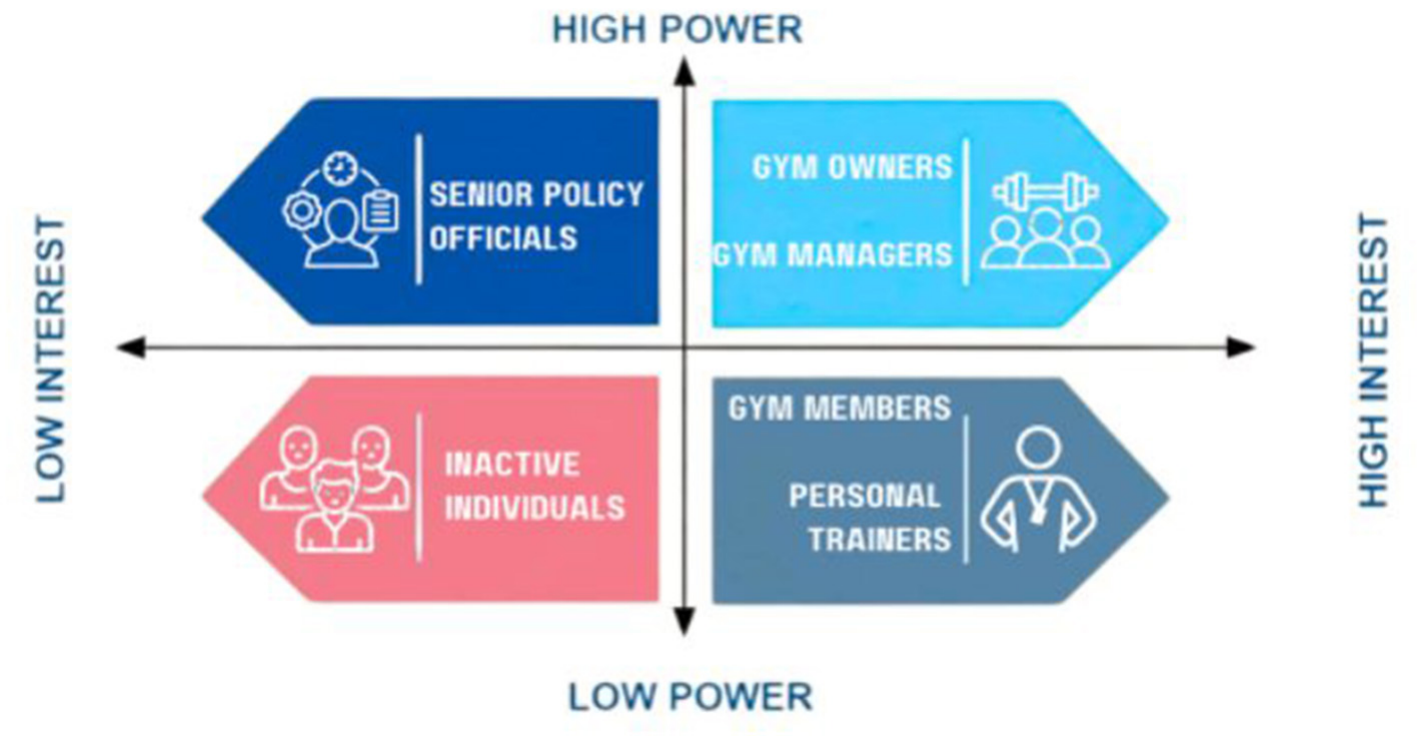
Mapping stakeholders in Dubai: power interest index.

These levels were then mapped onto the Power–Interest Matrix according to their relative influence (power) and engagement (interest) in the project, as illustrated in Figure 1.

This approach enabled strategic stakeholder mapping by aligning levels with engagement priorities. Stakeholder placement was informed by their role in decision-making, resource control, programme implementation, or behavioral impact. The classification supported tailored engagement strategies and effective allocation of communication efforts.

To ensure comprehensive representation across these stakeholder levels—ranging from senior policymakers to inactive individuals—a purposive sampling strategy was employed, supplemented by snowball and convenience sampling techniques. Initial participants were drawn from the fitness industry contacts known to the researcher, followed by targeted recruitment. The study adopted a broad definition of PA, encompassing exercise in gyms, fitness centers, sports clubs, homes, and outdoor environments, with recognition of Dubai's climate favoring indoor, climate-controlled settings.

According to Patton (18), determining sample size in qualitative research is guided by the study's aims, the depth of understanding sought, and practical considerations such as available resources and the richness of the data collected. In line with Sandelowski (19) and Vasileiou et al. (20), the sample was kept small to allow for in-depth, case-focused analysis. Malterud et al.'s (21) concept of “information power” informed the decision to recruit 2 to 4 participants per stakeholder group, as the focused research aim, and specificity of participants were expected to yield sufficiently rich data. The final sample included 20 participants (10 males and 10 females), aged 32 to 58, all Dubai residents. Gatekeeper approval was obtained for interviews with senior policy officials.

To reflect Dubai's multiethnic demographic, participants were selected from diverse nationalities, including Serbia, Iraq, the UK, the Philippines, Portugal, Azerbaijan, India, Pakistan, Canada, Syria, and Sri Lanka. A detailed summary of participant characteristics is provided in Appendix B.

Participants were selected based on their affiliation with chosen stakeholder groups and were Dubai residents. When approached, a total of 31 individuals responded and inquired about further details. They received participant information sheets (PIS) and consent forms via email, 20 of them agreed to go ahead with the interview, 2 individuals provided delayed responses, while the remaining recipients did not respond.

### Ethics

The researcher obtained informed consent from interviewees and recorded verbal consent from those who could not provide a written form. The London Sport Institute Middlesex University Ethics Sub-committee, and the Middlesex University Dubai, Research Committee approved the research. Stakeholder analysis involves ethical and practical issues like confidentiality. To ensure anonymity, identities were substituted with pseudonyms throughout the analysis. A significant challenge during semi-structured interviews was ensuring the neutrality of questions, especially when addressing spontaneous or unscripted follow-ups. To maintain research quality, researchers must craft questions carefully and be aware of potential sources of bias (22). The interview preparation aligned with Gubrium et al. (23) guidelines, emphasizing building trust, formulating insightful inquiries, and creating a supportive atmosphere.

The author's extensive 20-year residency and professional experience in the UAE, combined with cultural origins in the Arab world, provided insider perspective essential for understanding local PA practices and institutional dynamics. This positioning facilitated participant access and trust-building while enabling culturally informed data interpretation. To address potential insider bias, systematic reflexivity measures were implemented, including research journaling, peer debriefing sessions, and participant validation of findings, thereby strengthening the study's credibility and analytical rigor (24).

## Data collection

Semi-structured interviews (25) were conducted both face-to-face and online at participants' residences or workplaces, with each session lasting between 30 and 60 min. Most interviews took place online, offering greater scheduling flexibility and accommodating participants' availability. The interviews explored various aspects of PA interventions, including factors that facilitate or hinder engagement, common health concerns, and strategies for addressing cultural influences on health behaviors and lifestyle choices. The semi-structured format allowed the interviewer to tailor questions to each participant's background and context, ensuring relevance and depth in every conversation. This flexibility enabled the interviewer to prioritize prompts according to participants' lived experiences and roles, thereby enriching the quality of the data collected. At the same time, the approach maintained a consistent framework across interviews, allowing for systematic exploration of emerging themes.

### Interview guide development

The interview guide was constructed by first reviewing the study's theoretical framework and relevant literature on PA promotion and MetS. Questions were designed to elicit detailed responses about experiences, opinions, and strategies, in line with the study's qualitative approach. The draft interview guide was piloted with two volunteers unaffiliated with the final study group. Feedback was gathered regarding clarity, comprehensiveness, and relevance. Based on this input, as well as comments from supervisors and a qualitative research expert, the guide was revised to enhance its clarity and ensure comprehensive coverage of all intended themes. The final version was approved for face validity before interviews commenced.

The full set of interview prompts and questions used in this study is provided in Appendix A for reference.

## Data analysis

**Figure 2:**
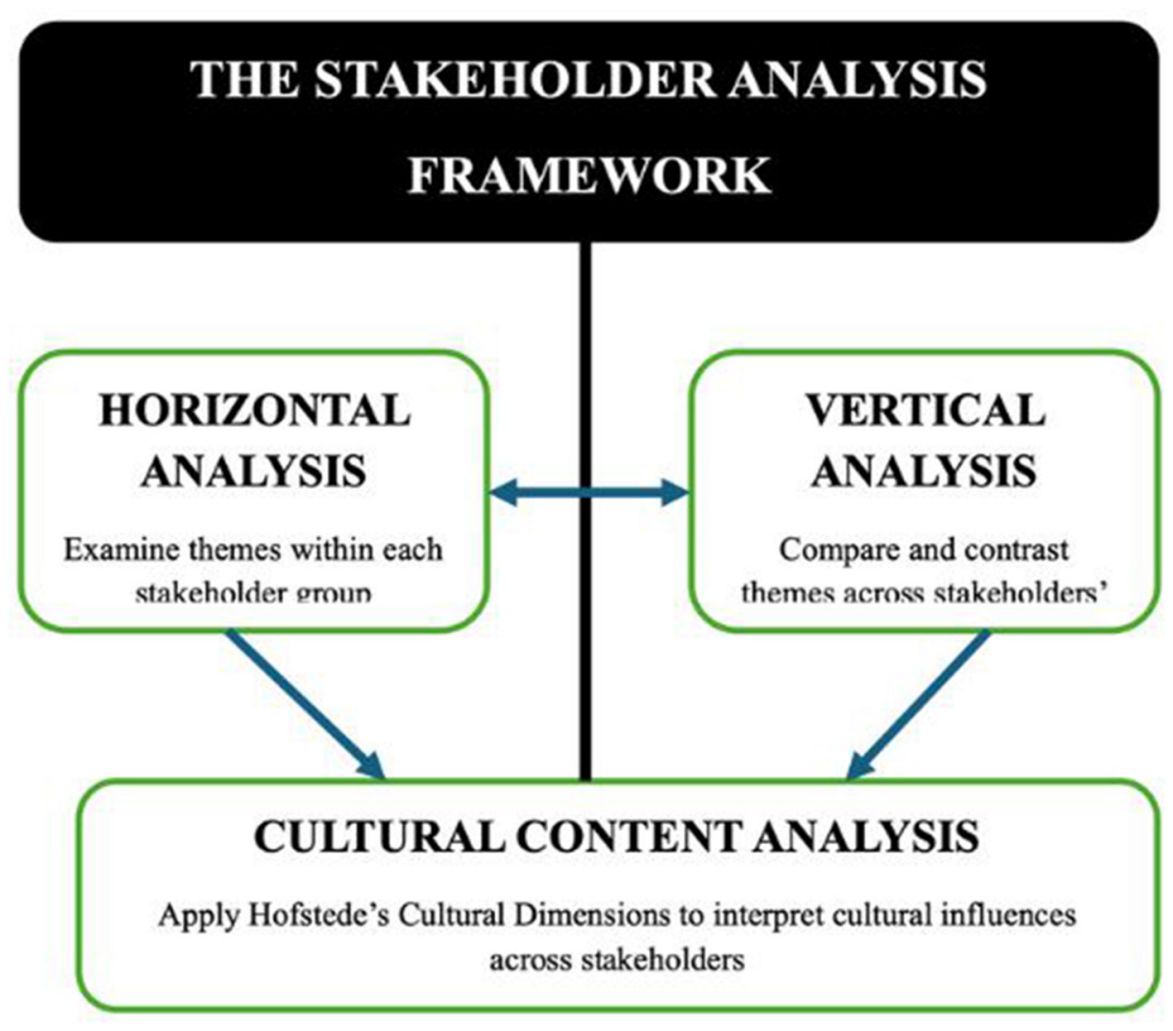
) The stakeholder analysis framework. A schematic overview of the three-layered analytic approach: horizontal analysis (within-group), vertical analysis (cross-group), and cultural content analysis using Hofstede's dimensions.

The qualitative data were analyzed using a multi-layered stakeholder analysis framework, which comprised horizontal analysis (within-group thematic analysis), vertical analysis (cross-group comparative analysis), and cultural content analysis using Hofstede's Cultural Dimensions (26). This framework enabled a systematic and nuanced exploration of stakeholder perspectives and cultural influences on PA participation in Dubai (see Figure 2).

### Data preparation and coding

Audio recordings were transcribed verbatim and anonymized. Initial coding involved manual identification of relevant passages and documentation of potential themes and categories. Subsequently, NVivo 12 (QSR International, Burlington, MA, USA) was used to facilitate electronic coding, organization, and retrieval of data. Both open (inductive) and hierarchical (deductive) coding approaches were applied, allowing for the emergence of new themes while also categorizing data according to stakeholder levels. All interviews, regardless of the specific prompts used, were thematically analyzed against the full set of research questions to maintain analytic consistency and comparability across participants.

### Horizontal analysis (within-group thematic analysis)

A preliminary horizontal analysis was conducted to identify recurring themes, patterns, and shared characteristics among stakeholders within the same group. This process enabled the recognition of experiences and attitudes unique to each stakeholder category.

### Vertical analysis (cross-group comparative analysis)

Following horizontal analysis, a vertical analysis was performed to examine variations in themes and perspectives across different levels of the stakeholder hierarchy. This involved comparing codes and themes across groups, from senior policy officials to inactive individuals, to reveal how organizational roles and positions influenced attitudes and behaviors related to PA. Hierarchical coding in NVivo supported this process, and themes were continuously refined through team discussions.

### Cultural content analysis

To assess cultural influences within Dubai's multi-ethnic population, a targeted content analysis was conducted using Hofstede's Cultural Dimensions Framework (26). Themes and codes related to cultural values, norms, and behaviors were mapped to established dimensions such as individualism vs. collectivism and power distance. This facilitated cross-cultural comparisons and provided deeper insight into how cultural context shapes stakeholder perspectives on PA. The use of Hofstede's framework aligns with its application in workplace wellness and global health initiatives, supporting the development of culturally tailored interventions.

### Integration of analytical methodologies

The integration of manual and electronic coding, horizontal and vertical analyses, and cultural content analysis enabled a thorough examination of stakeholder perspectives and the identification of cultural determinants influencing PA participation. NVivo's visualization and matrix query tools were used to explore relationships between codes and support robust theme development.

### Rigor and reflexivity

All coding and theme development were conducted in an iterative and reflexive manner, with regular team meetings to refine categories and resolve discrepancies. This ensured analytic rigor and credibility of findings.

## Findings

Stakeholder interviews revealed four key themes on the role of PA in addressing MetS in Dubai: (1) Gaps in Interventions and Awareness, (2) Barriers to PA, (3) Cultural Influences, and (4) Motivators and Opportunities. Although the interview guide included prompts about COVID-19′s impact, these responses did not contribute novel themes relevant to the study aims; thus, they are not reported in the results. Table 1 presents the themes, sub-themes, and representative quotations that illustrate stakeholder perspectives and support the thematic analysis.

**Table 1:** Themes, subthemes, and representative quotations from stakeholder interviews on the role of physical activity in addressing metabolic syndrome in Dubai.

**Theme 1**		
Gaps in Interventions and Awareness	Targeted awareness initiatives for women	“*Efforts have been dedicated to enhancing awareness of PA among women.”* (SPO-02)
	Lack of awareness about MetS	“*My husband has all of that. I have a cholesterol issue also, but with medicines, our numbers are in control”* (INAC-02)
**Theme 2**		
Barriers to PA	Focus on body image over holistic health	“*At least 9 out of 10 inquire for help to lose belly fat”* (PT-01)
	Lack of time due to competing priorities	*I couldn't attend the event, we planned actually, my friends went over there when they were having an off day. But I was really busy in those days of school (work), so I couldn't manage to…”* (INAC-03)
	Life-work balance	“*Yesterday I finished the work at 3:30 (am) and woke up today at 8(am), and I skipped my exercise class, which means I have to walk in the evening”* (GMEM-01)
	Life-work balance	*Last year it was like that (I was active) but this year, I just haven't pushed myself enough, I don't know why but maybe again this has been a busy year for us at work so there's a lot of that going on. Interrupted by phone calls in the middle (of workout) then you stop and then you just don't want to get back in.”* (INAC-01)
	Sedentary behavior	“*When I meet clients who say that they only work out thrice a week and do nothing else, I tell them, unfortunately, it doesn't matter if you sit down the whole day and then do 1 h in the gym, it will not make a deal, it won't be beneficial.”* (PT-02)
	Information management under temporal pressure	“*It's actually exciting that the municipality is really proud of them (the number of parks) and so they should be too, and most of those parks are free to participate, there's always initiatives going on. So that's not a barrier… but people's time and a little bit of sort of information confusion and overload I think is holding people back a bit.”* (GO-02)
	Cultural variation in PA awareness and motivation	“*Europeans have like very high awareness of the benefits of physical activity, whereas Arabic nationalities are becoming more progressively aware, and they are relying on the physical activity, I can see that boom and that progressive engagement. And then when you go to the more Asian area, they have like the minimum commitment to that, and still, they are not willing to accept that investment in their own health, it's more about obligation related with the price”*. (GO-03)
	Lack of guidance and support in fitness settings	“*So, they don't guide you properly if you don't have a one-to-one session with them. Sometimes I have seen people struggling with machines. I don't use machines that much, so I don't know how to use them properly, I am always looking for help, but they (personal trainers) don't guide you the way they are supposed to”. (GMEM-03)*
**Theme 3**		
Cultural dimensions and their influence	High power distance index	“*It's hard to make her (mother-in-law) understand that I need to take out this time for the gym, she thinks I enjoy going there, that it's my time off but its not, I need to, but I can't argue with her on that, it's no use”* (GO-03)
	Collectivism	“*I never went on a desert hike or a desert activity with my parents, we were not that type of family, just the typical Syrian Arabic ex-pat in UAE. Its activities were all about barbecues and dinners and luncheons and visiting friends and you know, NO NO physical activity.”* (GM-01)
**Theme 4**		
Motivators and Opportunities	Enjoyment and engagement	“*The first week was so hard I used to tie a towel on my waist because it used to hurt me so much and then, but I didn't give up because I somehow liked the whole theme of this ‘dance workout', the music, the choreography”*. (GMEM-02)
	Infrastructure and variety	“*An extensive collection of 56 varied sports, encompassing activities such as Padlet, Kabaddi, Fencing, Jiujitsu, and others, has been introduced. A project is underway to construct the world's largest cycling track spanning 400–500 kilometers”* (SPO-02)
	Supportive environment	“*If you want to have physical activities outside of your home, I don't see any barriers as such, because we have a lot of facilities, there are so many good fitness centers, the parks here are so scenic, the winter months here are so beautiful, and I live in a gated community where they have this beautiful walking parks.”* (GO-01)

SPO, Senior policy officials; GO, Gym Owner; GM, Gym manager; PT, Personal trainer; GMEM, gym members; INAC, inactive individuals.

## Results

### Gaps in interventions and awareness

Participants widely recognized the presence of PA initiatives and facilities across Dubai, reflecting government and community efforts to promote active lifestyles. However, many expressed concerns about the inclusivity and accessibility of these programs, suggesting that certain population groups—such as lower-income individuals or culturally diverse communities—may be underserved. Several participants noted that while facilities exist, their geographic distribution and operating hours do not always align with users' needs, limiting practical access.

A recurrent theme was the lack of sustained engagement; participants observed that initial enthusiasm often waned due to insufficient follow-up or program adaptation over time. The absence of culturally tailored programming was also highlighted, with some reporting that interventions failed to reflect the social norms or preferences of their communities, thereby reducing relevance and appeal.

A critical finding was the pervasive low awareness of MetS and its link to PA. Many participants demonstrated limited understanding of how regular PA can prevent or manage metabolic conditions, frequently associating treatment primarily with medication. This knowledge gap appeared to reduce the urgency for adopting preventive behaviors like exercise, resulting in inconsistent or minimal participation. Furthermore, even those motivated to engage with PA resources encountered barriers such as unclear messaging or inadequate support, which hindered effective utilization of existing interventions.

### Barriers to PA

Multiple barriers to PA were reported, each reflecting different dimensions of participants' lived experiences. Financial constraints were a prominent obstacle, with several individuals highlighting the prohibitive cost of gym memberships or specialized programs. This economic barrier was particularly acute for lower socioeconomic groups, restricting their options for structured exercise.

Within fitness facilities, participants described a lack of adequate guidance and personalized support. Many recounted experiences where personal trainers prioritized sales targets over providing foundational instruction or encouragement, leaving members feeling unsupported. Some trainers themselves acknowledged institutional pressures to focus on client acquisition, which limited their ability to assist non-paying members effectively.

Time scarcity emerged as a significant barrier, with participants balancing demanding work schedules, family responsibilities, and social commitments. The challenge of fitting regular exercise into busy days was a common refrain, often resulting in sporadic or insufficient activity. Additionally, extended periods of sedentary behavior during work hours were noted as a concern, with participants recognizing that occasional exercise did not fully offset prolonged inactivity.

### Cultural dimensions influencing PA

Cultural context played a central role in shaping PA behaviors. In environments characterized by high Power Distance Index (PDI), participants reported limited capacity to advocate for their own needs regarding exercise time or facilities, particularly in workplace and family settings. Deference to authority figures often constrained grassroots initiatives but simultaneously elevated the influence of formal prescriptions, such as medical advice, which could legitimize PA within hierarchical social structures.

Collectivist values were evident in participants' prioritization of family and community obligations over personal exercise routines. Many described feeling compelled to attend traditional events or fulfill social roles that took precedence over PA, complicating efforts to maintain consistent activity. These cultural expectations often reduced individual autonomy in health-related decision-making.

Awareness and motivation levels varied significantly across cultural groups. While some demonstrated clear understanding and intrinsic motivation for PA, others participated primarily due to external pressures or social expectations. This heterogeneity underscored the complexity of cultural factors influencing behavior.

### Motivators and opportunities

Despite barriers, many participants exhibited resilience and adaptability in sustaining PA. Enjoyment was frequently cited as a powerful motivator, with dance-based workouts and group activities fostering both physical engagement and social connection. Participants expressed pride in recent community infrastructure developments, such as new sports facilities and cycling tracks, which enhanced accessibility and created opportunities for shared experiences.

The social dimension of PA was important; activities that promoted community belonging and were perceived as enjoyable helped overcome initial discomfort or logistical challenges. Participants also valued accessible guidance and supportive environments, which contributed to sustained participation.

### Stakeholder perspectives

The study's stakeholder-driven design captured diverse perspectives from multiple organizational levels and cultural backgrounds. This inclusivity provided a nuanced, multi-ethnic understanding of factors influencing PA engagement, enhancing the relevance of findings to Dubai's heterogeneous population.

Overall, these findings align closely with the research aims by providing a detailed and comprehensive understanding of the systemic, cultural, and individual factors influencing PA engagement for MetS prevention in Dubai. This alignment confirms that the study effectively captured the key dimensions it intended to investigate.

## Discussion

This study provides valuable insights into the complex landscape of PA engagement as a strategy for preventing MetS in Dubai. The findings highlight critical gaps in awareness and intervention design, systemic and cultural barriers, as well as motivators and opportunities that shape PA participation.

The pervasive lack of awareness about MetS and its relationship to PA is a significant concern. Participants' limited understanding aligns with prior research emphasizing the need for effective translation of scientific knowledge into accessible public health messaging (27). This gap undermines motivation for preventive behaviors and suggests that existing health promotion efforts may not be sufficiently targeted or comprehensible to diverse populations.

Financial and structural barriers identified reflect well-established socioeconomic determinants of PA participation (28, 39)). The reported inadequacy of guidance and personalized support within fitness environments parallels findings from Bauman et al. (29), underscoring how service delivery models focused on sales rather than member support can discourage sustained engagement.

Time scarcity and concerns about prolonged sedentary work patterns further complicate PA adherence, consistent with literature documenting these as common obstacles (30, 31). These findings support calls for interventions that not only promote structured exercise but also encourage frequent movement throughout the day, such as active breaks or ergonomic workplace adjustments.

Cultural factors emerged as pivotal determinants. High Power Distance Index (PDI) contexts limit individual agency but enhance the authority of healthcare providers, whose recommendations carry substantial weight and can legitimize PA within hierarchical and collectivist family structures (26, 32). The collectivist emphasis on communal obligations consistently took precedence over individual health pursuits, mirroring findings across diverse populations (29, 33, 34).

These cultural dynamics underscore the importance of tailored, culturally sensitive interventions. Integrating PA into communal events or offering family-oriented exercise options may better align with local values. Framing PA as beneficial to the collective and endorsed by authoritative medical advice can enhance acceptance and adherence (35, 36).

The strong role of enjoyment and community infrastructure as motivators aligns with self-determination theory, which identifies intrinsic motivation as critical to sustained PA (37). Participants' positive response to engaging formats and social connectedness suggests that interventions prioritizing these aspects may be particularly effective (38).

Finally, the stakeholder-driven and culturally inclusive design of this study strengthens the applicability of findings across Dubai's multi-ethnic population and offers valuable lessons for other rapidly urbanizing, multicultural settings facing similar challenges.

In conclusion, the results and their interpretation align with the research aims by elucidating the multifaceted interactions among policy, culture, and individual behavior that shape PA participation. This alignment highlights the study's contribution to advancing culturally informed and contextually relevant strategies for MetS prevention.

## Broader implications

The findings of this study emphasize the critical need for public health interventions that are not only comprehensive and sustainable but also deeply attuned to the cultural, structural, and socioeconomic realities of multi-ethnic urban environments such as Dubai. Generic or one-size-fits-all health promotion messages are unlikely to achieve the desired impact without addressing the specific barriers and motivational drivers identified here.

Integrated, cross-sectoral collaboration emerges as a key strategy. Healthcare providers, who hold respected positions especially in high PDI societies, should play a central role in promoting PA. Their involvement as trusted sources of culturally sensitive health education and advocates for active lifestyles can legitimize PA behaviors, enabling individuals to negotiate time and space for exercise within hierarchical family and social structures.

Partnerships that bridge healthcare systems, fitness professionals, and community organizations are essential to create continuity of care and foster sustained engagement. Fitness environments must evolve to provide culturally competent support and personalized guidance rather than prioritizing sales targets. This approach can help overcome barriers related to inadequate assistance and enhance long-term participation.

Tailored educational initiatives co-developed with community leaders and healthcare professionals can effectively bridge knowledge gaps and accommodate diverse cultural values. Strategies that integrate PA into communal events or family-oriented activities will resonate better with collectivist populations and improve relevance and uptake.

These insights have broader relevance beyond Dubai. Other rapidly urbanizing cities with diverse populations facing metabolic health challenges can draw upon these findings to develop culturally grounded, medically integrated strategies to combat sedentary lifestyles and improve metabolic health outcomes. In summary, the results, themes, and their interpretation align with the research aims by providing nuanced insights into how policy, culture, and behavior interact to shape PA participation, thereby informing culturally appropriate strategies to address MetS.

## Limitations

This study has several limitations that should be considered. First, the voluntary nature of participation may have introduced self-selection bias, potentially limiting the representativeness of the sample and the generalizability of the findings. It is possible that individuals with a greater interest or experience in PA were more likely to participate. Second, time constraints and limited access to senior stakeholders may have impacted the depth and breadth of data saturation, restricting the exploration of certain perspectives or themes. Third, regional skepticism toward research and concerns about confidentiality could have influenced participants' openness, potentially resulting in socially desirable responses, particularly when discussing cultural or institutional barriers. Fourth, while the semi-structured interview format provided valuable flexibility, it also introduced variability in data collection that may affect the comparability of responses across interviews. Finally, although the interview guide included prompts regarding the impact of COVID-19, participant responses did not yield distinct or relevant themes aligned with the study's aims. Consequently, these data were excluded from the main findings, which may limit the comprehensiveness of pandemic-related influences in our results. Future research should seek to address these limitations through larger, more representative quantitative studies and the use of mixed methods approaches to validate and expand upon these findings.

## Conclusion

This research offers a comprehensive exploration of the multifaceted interplay between policy, culture, and individual behavior influencing PA engagement as a strategy to address MetS in Dubai. By capturing diverse stakeholder perspectives, from policymakers to less active individuals, the study reveals both encouraging progress and persistent barriers.

Despite strong policy commitments and infrastructural developments, financial constraints, limited public awareness, and entrenched sedentary cultural norms continue to challenge widespread PA participation. The nuanced examination of cultural factors, particularly high PDI and collectivist values provides actionable insights for tailoring interventions that resonate locally.

These findings establish a foundation for developing culturally sensitive, evidence-based strategies aligned with Dubai's vision of becoming a global leader in fitness and reducing the metabolic disease burden. Moreover, the lessons learned here offer valuable guidance for public health efforts in other rapidly urbanizing, multicultural settings.

Sustained investment in culturally responsive interventions, ongoing research, and cross-sectoral collaboration will be essential to advance PA promotion and improve metabolic health outcomes both locally and globally.

## Data Availability

The datasets generated and analyzed during the current study contain interview transcripts in which all participant identifiers have been replaced with pseudonyms to preserve confidentiality. Due to the sensitive nature of the qualitative data, and in accordance with participant consent and ethical guidelines, the full transcripts are not publicly available. Data may be made available from the corresponding author on reasonable request, subject to ethical approval and in line with data protection laws.
