# Supplementary material for: Perspectives of physical activity in combating metabolic syndrome: insights from a multi-ethnic urban population
Source: Front Public Health. 2025 Aug 22;13:1477025. doi: 10.3389/fpubh.2025.1477025 (PMC12412454; doi:10.3389/fpubh.2025.1477025)
Supplement: Supplementary file 1 [file Data_Sheet_1.pdf]

Characteristic-Category-n-

Characteristic	Category	n	%
**Total Participants**		20	100
**Gender**	Male	10	50
	Female	10	50
**Age (years)**			
	30–39	7	35
	40–49	8	40
	50- 59	5	25
**Nationality**	UAE	3	15
	India	4	20.0
	Serbia	2	10
	Pakistan	2	10
	Canada	1	5
	Azerbaijan	1	5
	Iraq	1	5
	Syria	1	5
	Phillipines	1	5
	Portugal	1	5
	Srilanka	1	5
	UK	2	10
**Stakeholder Level**	Senior Policy Officials	2	10
	Gym Owner	4	20
	Gym manager	3	15
	Personal Trainers	3	15
	Gym members	4	20
	Inactive individuals	4	20

In the qualitative findings and thematic analysis, participants are referred to using anonymized IDs based on their stakeholder group. Each ID consists of a group-specific prefix followed by a number (e.g., *PT01* = Personal Trainer 1). The stakeholder group prefixes are as follows:

- **SPO –** Senior Policy Official
- **GO –** Gym Owner
- **GM –** Gym Manager
- **PT –** Personal Trainer
- **GMEM –** Gym Member
- **INAC –** Inactive Individual

These IDs are used throughout the findings section to attribute quotations and insights while maintaining participant confidentiality.
