# Supplementary material for: Perspectives of physical activity in combating metabolic syndrome: insights from a multi-ethnic urban population
Source: Front Public Health. 2025 Aug 22;13:1477025. doi: 10.3389/fpubh.2025.1477025 (PMC12412454; doi:10.3389/fpubh.2025.1477025)
Supplement: Supplementary file 2 [file Table_1.docx]

**Appendix: Semi-Structured Interview Guide**

**Introduction and Consent**

Thank you for agreeing to take part in this interview. We are speaking with you today to better understand the experiences, views, and needs of Dubai residents regarding physical activity, health, and related interventions—especially in the context of chronic diseases. Our goal is to learn from your perspective so we can improve the design and delivery of physical activity programs and health support in our community.

There are no right or wrong answers. We are interested in your honest opinions and personal experiences.

Participation in this study is entirely voluntary. Your choice to participate, decline, or withdraw at any time will not affect any services or programs you currently receive. The interview will take about 30-60 minutes, but you are free to share as much or as little as you wish.

With your permission, I would like to audio record our conversation to ensure that I capture your responses accurately. All information you provide will be kept confidential. Your responses will be de-identified and only shared with the research team. Nothing you say will be linked to your name or identity in any reports or publications.

You may skip any question or stop the interview at any time, for any reason.
Do you have any questions about what I have just explained?
May I record our conversation for accuracy?

**Section 1: Background and Demographics**

1. Can you tell me a little about yourself, your current role, and how long you have lived in the UAE?
2. What is your involvement with physical activity, either personally or professionally?

**Section 2: Impact of COVID-19**

1. How did the COVID-19 pandemic affect your lifestyle or that of those close to you, particularly regarding physical activity?
2. Have you observed any lasting changes in physical activity patterns since the pandemic?

**Section 3: Physical Activity Interventions**

1. The Dubai Fitness Challenge (30x30) recently concluded. Did you participate or contribute to this initiative? If so, in what capacity?
2. In your opinion, what makes an intervention to increase physical activity effective in this context?

**Section 4: Barriers and Facilitators**

1. What do you see as the most significant barriers to physical activity for residents in Dubai?
2. What strategies do you think could help overcome these barriers?
3. What factors do you believe help facilitate higher levels of physical activity in the community?

**Section 5: Metabolic Syndrome and Health**

1. Are you familiar with metabolic syndrome and its link to non-communicable diseases?
2. What are the most common health issues you observe among your family, friends, or clients?
3. Do you work with individuals affected by metabolic syndrome? Are there specific programs or professionals dedicated to supporting them?

**Section 6: Cultural and Social Diversity**

1. Dubai is home to a highly diverse population. In your view, is there a need for culturally tailored interventions to promote physical activity?
2. What changes have you noticed in the population’s approach to physical activity as diversity increases?
3. What challenges does this diversity present to your organization or community?
4. How does your organization demonstrate its commitment to cultural diversity and inclusion?
5. What measures are in place to accommodate differences in values, norms, and practices?
6. If no action is taken to address diversity and inclusion, what do you think the impact would be?
7. Which ethnic groups do you perceive as most and least active, and why?
8. Are there specific social or cultural barriers that affect participation in physical activity? What steps have been taken to address these?

**Section 7: Personal Experience and Motivation (For Gym and Non-Gym Members)**

1. How do you feel about exercising in general?
2. How confident are you in your ability to exercise regularly?
3. What factors influenced your decision to join (or not join) a gym?
4. Do you feel your expectations regarding physical activity have been met?
5. Have you noticed any changes in your exercise habits over time?
6. Have there been occasions when you chose not to exercise? If so, what were the reasons?

**Closing**

1. Is there anything else you would like to add or any final thoughts you wish to share?
2. Would you like to receive a summary of the study findings?

This interview guide was used as a flexible framework for semi-structured interviews. Not all questions were asked in every interview. The guide served as a prompt, and the interviewer adapted the questions, and their order based on the flow of conversation and the relevance to each participant’s background and experiences.
